# OpenEDS2020 Challenge on Gaze Tracking for VR: Dataset and Results

**DOI:** 10.3390/s21144769

**Published:** 2021-07-13

**Authors:** Cristina Palmero, Abhishek Sharma, Karsten Behrendt, Kapil Krishnakumar, Oleg V. Komogortsev, Sachin S. Talathi

**Affiliations:** 1 Department of Mathematics and Informatics, Universitat de Barcelona, 08007 Barcelona, Spain; crpalmec7@alumnes.ub.edu; 2 Computer Vision Center, Campus UAB, 08193 Bellaterra, Spain; 3 Eye Tracking Department, Facebook Reality Labs Research, Redmond, WA 98052, USA; abhishek.sharma@fb.com (A.S.); ok1@fb.com (O.V.K.); 4 Facebook Reality Labs, Menlo Park, CA 94025, USA; karsten@fb.com (K.B.); kapilk@fb.com (K.K.); 5 Department of Computer Science, Texas State University, San Marcos, TX 78666, USA

**Keywords:** gaze prediction, semantic segmentation, gaze estimation, video oculography, virtual reality

## Abstract

This paper summarizes the OpenEDS 2020 Challenge dataset, the proposed baselines, and results obtained by the top three winners of each competition: (1) Gaze prediction Challenge, with the goal of predicting the gaze vector 1 to 5 frames into the future based on a sequence of previous eye images, and (2) Sparse Temporal Semantic Segmentation Challenge, with the goal of using temporal information to propagate semantic eye labels to contiguous eye image frames. Both competitions were based on the OpenEDS2020 dataset, a novel dataset of eye-image sequences captured at a frame rate of 100 Hz under controlled illumination, using a virtual-reality head-mounted display with two synchronized eye-facing cameras. The dataset, which we make publicly available for the research community, consists of 87 subjects performing several gaze-elicited tasks, and is divided into 2 subsets, one for each competition task. The proposed baselines, based on deep learning approaches, obtained an average angular error of 5.37 degrees for gaze prediction, and a mean intersection over union score (mIoU) of 84.1% for semantic segmentation. The winning solutions were able to outperform the baselines, obtaining up to 3.17 degrees for the former task and 95.2% mIoU for the latter.

## 1. Introduction

Eye tracking has emerged as a powerful tool for several applications, including health assessment and intervention [1,2], and human behavior and communication analysis [3,4]. Nonetheless, the fields that have recently boosted its potential are virtual reality (VR) and augmented reality (AR). Indeed, the potential applications of AR/VR technology to a multitude of sectors such as online education [5], healthcare [6,7], entertainment [8,9], communication [10,11] and/or gaming industry [12,13] have created an ever-growing demand for more realistic and immersive AR/VR experiences.

One of the core technologies that enables high-quality immersive VR/AR experiences while keeping a low computational cost of rendering the environment is called Foveated Rendering (FR) [14]. FR renders a high-quality picture at the point where a user is looking, while reducing the quality of the picture in the periphery according to a function of human visual acuity. This non-uniform image degradation substantially reduces the graphical pipeline’s power consumption without decreasing the perceptual quality of the generated picture. Thus, to maintain an optimal user experience, it is crucial for FR to have low-latency, high-accuracy gaze estimates, which is also a desired feature for other eye tracking applications such as gaze-based interaction.

Solutions for image/video-based gaze estimation, also known as *video oculography*, can be broadly classified into geometric and appearance-based systems [15]. Geometric approaches treat the human eye as a sphere-on-sphere model [16], and find the pupil and glint (Here we use word "glint" rather than 1st Purkinje image. Although, theoretically, the capture of other Purkinje images is possible [17], it is an extremely unlikely occurrence in any non-specialized video oculography system. In the setup employed in this work only 1st Purkinje images are detected and are referred as glints.) locations in a precisely calibrated system of cameras with infrared (IR) illumination and LEDs to infer the 3D location of the pupil and the gaze direction. More recent geometric approaches do not require dedicated systems and/or glints, and make use of 3D morphable models instead [18,19] with off-the-shelf RGB cameras. On the other hand, appearance-based models are typically based on end-to-end inference models, such as deep Convolutional Neural Networks (CNNs) [20,21], to directly estimate the gaze direction in the frame of reference of the camera. Presently, both geometric and appearance-based approaches usually rely on one or more deep learning modules to tackle the large variations in eye appearances due to anatomical differences, lighting, camera viewpoint, use of glasses and/or makeup across the human population. Whereas current state-of-the-art appearance-based methods exploit end-to-end deep networks to directly regress gaze from input eye/face images, geometric methods deploy highly accurate per-pixel (also known as semantic) segmentation networks to extract key eye regions (namely pupil, iris, sclera, and everything else, also referred to as background) for further processing [22].

In real-world applications, the input to any gaze estimation system is temporal sequences of eye images in the form of a video. However, most popular approaches do not exploit temporal information and instead estimate gaze direction for each frame separately. Intuitively, there exists useful temporal information in videos that can be leveraged to improve current gaze estimation approaches, for example, by incorporating spatio-temporal features captured over a sequence of eye images [23]. Similarly, spatio-temporal modeling of the eye movements can be leveraged to propagate the segmentation mask from a handful of frames to a complete sequence for yielding large amounts of high-quality labeled data for training segmentation networks at a fraction of the per-frame annotation cost and time. Several works have recently started to exploit this area of research [24,25] using datasets based on low resolution/sampling rate, remote RGB cameras (e.g., EYEDIAP [26], EVE [27]). However, there is a lack of high-resolution, real-world datasets with images sampled at a sufficient sampling rate to capture trajectories of such fast eye movements as saccades and with accurate ground-truth annotations to advance research on the topic.

**Figure 1:**
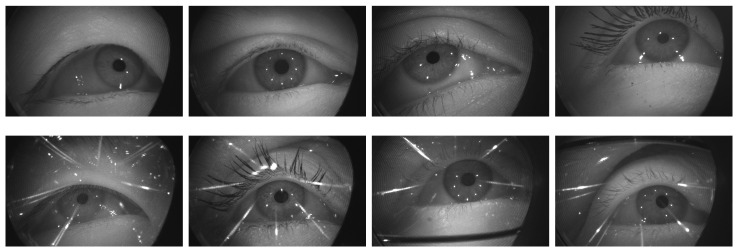
Examples of images without glasses (**top row**) and with glasses (**bottom row**), representing the variability of the dataset in terms of accessories, ethnicity, age and gender.

Motivated from the aforementioned gap in the literature, we organized the OpenEDS 2020 Challenge, the second iteration of the OpenEDS-Eye Tracking for VR and AR Challenges, to foster research on spatio-temporal methods for gaze estimation, gaze prediction, and semantic segmentation. In particular, the challenge was divided into 2 competition tracks: (1) Gaze Prediction, which called for designing solutions to predict future gaze directions by leveraging the spatio-temporal information encoded in a sequence of recorded eye images; and (2) Sparse Temporal Semantic Segmentation, which called for designing solutions to propagate semantic segmentation labels for key eye regions, by leveraging a few hand-labeled frames in a given video sequence of eye images. Both tracks were based on a novel dataset, OpenEDS2020, a large-scale anonymized dataset of sequences of high-resolution eye images (640 × 400 pixels) sampled at 100 Hz with semantic segmentation and gaze direction annotations, which features up to 87 subjects with a wide range of appearance variability. The dataset was collected using a VR head-mounted display (VR-HMD) equipped with two synchronized eye-facing IR cameras. Example images of the dataset are shown in Figure 1.

In this paper, we describe the rationale behind both challenges, the methodological baselines used, and solutions proposed by the challenge winners of both competition tracks. In addition, we describe in detail the collection and curation process of the OpenEDS2020 dataset, which we publicly release to the research community. We believe this dataset can be instrumental in advancing the current state of the art in gaze estimation, gaze prediction, and semantic segmentation research, and benchmarking of existing or future algorithms.

The remainder of this paper is organized as follows. Section 2 introduces the challenge objectives and evaluation protocol used. Section 3 describes the OpenEDS2020 dataset. Section 4 describes the methodological baselines used for the challenge. Section 5 summarizes the solutions proposed by the top-3 winners of each competition. Finally, Section 6 concludes the paper.

## 2. OpenEDS 2020 Challenge: Description

The OpenEDS 2020 Challenge (https://research.fb.com/programs/openeds-2020-challenge/, accessed on 25 June 2021) was hosted as part of the *OpenEyes: Eye gaze in AR, VR and in the Wild* (https://openeyes-workshop.github.io/, accessed on 25 June 2021) workshop, organized at the European Conference on Computer Vision in 2020. The challenge was divided into 2 competition tracks: (1) Gaze Prediction Challenge, and (2) Sparse Segmentation Challenge. Both tracks were hosted on the Eval.ai (https://eval.ai, accessed on 25 June 2021) platform and were active from 1 April through 31 July 2020. To participate in either of the tracks, participants had to request access to the dataset and register in the submission platform. Their goal was to devise solutions for either or both challenge tracks using a defined training/validation set, apply their methods to a test set with hidden labels, and upload their predictions to the submission platform to obtain the performance score of the given solution. Such platform included a public leaderboard where participants could compare the performance of their solution against other teams and the baselines proposed by the organizers. The competition tracks and their evaluation protocol are described below.

### 2.1. Gaze Prediction Challenge

The Human Visual System exhibits a variety of eye movements—fixations, saccades, smooth pursuits, vestibulo-ocular reflex, vergence, and vestibular and optokinetic nystagmus [28]. Among above mentioned eye movements, saccades are the fastest with velocities reaching up to 700 deg/s, thus transitioning the eye from one point of fixation to the next very quickly and in a ballistic manner [28].

Various applications for eye tracking, such as FR and gaze-based interaction, benefit from low-latency gaze estimates. Fast eye movements such as saccades present a challenge for FR specifically, due to the transmission and processing delays present in the eye tracking and graphical pipelines. If such pipelines do not compensate for the delays, fast eye movements can take the user’s gaze to image areas that are rendered with low quality, thus degrading the user’s experience. Among the ways of remedying this issue are: a reduction of delays, which is not always possible; predicting future gaze locations, thus compensating for the delays; or a combination of both.

Prediction of future gaze locations can be accomplished based on previously estimated gaze points, understanding of the content of the presented scene (i.e., visual saliency), or a combination of both. Considering real-time requirements of FR and its goal of reducing power consumption, the prediction of future gaze points based on a short subsequence of the already-estimated gaze locations is considered the most fruitful path of exploration. If predicting future gaze locations with high accuracy is feasible, it would allow an implementation of an FR method that would match closely with the human visual acuity function. As a result, it could encode only a tiny part of the image at a high-quality resolution, providing the highest level of energy savings.

Instead of predicting gaze locations, for which the accommodation and vergence of the two eyes would need to be considered, the challenge focused on monocular 3D gaze direction prediction as a first step of a gaze location prediction approach. In particular, the challenge called for the following: (a) Predicting future gaze directions based on the previously estimated gaze direction vectors of the previous eye images; (b) Predicting future gaze directions by leveraging the spatio-temporal information encoded in the sequence of previous eye images.

The task consisted in designing a model to predict the 3D gaze direction vector up to 50 ms into the future, given a sequence of previous eye images. For a dataset recorded at 100 Hz, 50 ms is equivalent to 5 frames. Participants of the challenge were scored on a test set, which contained the sequence of previous eye images with hidden ground-truth vectors, using the performance metric of Prediction Error (PE), defined as:

(1) $$\mathrm{PE}=\frac{{\mathrm{PE}}_{t}}{5},\mathrm{where}{\mathrm{PE}}_{t}=\frac{\sum_{s}^{\left|S\right|}d(g_{t,s},{\hat{g}}_{t,s})}{\left|S\right|}\mathrm{for}t\in \left[1,5\right],$$

where |S| is the number of sequences in the test set, $g_{t,s}$ is the ground-truth 3D gaze direction vector at time t*10 ms after the last provided sample of the sequence *s*, and ${\hat{g}}_{t,s}$ is the corresponding gaze prediction. d(·) refers to the per-frame angular error between estimated and ground-truth gaze vectors, such that:

(2) $$d\left(g,\hat{g}\right)=\arccos\frac{g\cdot \hat{g}}{\left||g|\right|\cdot \left|\right|\hat{g}\left|\right|}.$$

The 3D gaze direction vector is defined as the 3D unit (normalized) vector in the direction of the visual axis, represented in the 3D Cartesian coordinate system. For training, participants were provided with both eye images and ground-truth vectors, so that they could train appearance-based gaze estimation models using the given dataset. No subject calibration details were provided.

### 2.2. Sparse Temporal Semantic Segmentation Challenge

Many eye tracking solutions require accurate estimation of eye features from 2D eye images. Typically, this is done via semantic segmentation of key eye regions. To generalize models for per-pixel segmentation of unseen eye images from a diverse population under different eye states (fully open, half-open, closed) and different makeup conditions, the model training stage requires large-scale, hand-annotated training data that can be costly and time-consuming. However, it is easy to obtain a data acquisition setup that captures medium-to-short duration (5–25 s) video sequences of eye images and manually label a handful of images (5%) for each sequence.

Then, the goal of the challenge was to solve label propagation with a limited number of samples per sequence. Solving this problem allows one to have a large set of annotations without spending large amounts of resources on human annotation. We posit that the small fraction of hand-annotated labels can be accurately propagated to the rest of the images in the sequence by leveraging temporal information along with geometric and photometric consistencies arising from the eye images of the same person. Such approaches called for innovative algorithms to leverage the aforementioned cues. Some promising directions were foreseen to be: (a) Temporal co-segmentation using deep learning; (b) Temporal few-shot learning framework; (c) Learning and respecting the natural representation, including the geometry, of human eyes for temporal label propagation; (d) Leveraging synthetic data generation if and where appropriate.

Participants of the challenge were given sequences of eye images with a small fraction of them annotated with semantic segmentation masks. The task was to design a model to perform semantic segmentation on a subset of the images that did not have an annotated mask, which we refer to as test subset. The performance metric used to evaluate the proposed solutions was the mean Intersection over Union (mIoU, also known as Jaccard index) over all the key regions of all the images conforming the test subset.

## 3. OpenEDS2020 Dataset

We believe that an eye tracking dataset designed for potential spatio-temporal methods should contain a sufficiently representative gaze angle distribution and appearance variability to train gaze estimation or semantic segmentation models, while ensuring variability in terms of eye movements, directions, and velocities to train prediction models.

Due to the complexity of annotating accurate gaze direction and eye movements, there is a limited number of publicly available datasets of eye-image sequences with high appearance diversity providing multiple annotations (see Table 1 for a summary). In this section, we introduce the OpenEDS2020 dataset, a novel dataset of eye-image sequences from 87 subjects captured at a frame rate of 100 Hz under controlled illumination, using a VR-HMD. In contrast to other existing datasets, our dataset provides the highest number of subjects, with a frame rate sufficient to capture different eye movement types (fixations, saccades, and smooth pursuit).

The dataset, which we are releasing to the broader research community, is divided into 2 subsets, one for each competition track of the challenge. Although there is an overlap between the subjects included in each subset (67 out of 87 appear in both subsets), the recordings from which the eye-image sequences are obtained are unique to each subset. Apart from the data used in the challenge, the new public version of the dataset is enriched with additional per-image/per-sequence annotations. The data collection procedure, curation process of each data subset, and annotations provided are described below.

### 3.1. Data Collection

The dataset was captured from 90 voluntary subjects of ages between 20 and 70 years old with appearance variability in terms of ethnicity, gender, and age, and some of them wearing glasses, contact lenses, and/or makeup. There was no assessment by an eye health professional or cut-off by visual performance. These subjects were all recruited internally, and provided written informed consent for using their eye images for research and commercial purposes before taking part in the data collection stage. Subjects were asked to wear a VR-HMD, mounted with two synchronized eye-facing IR cameras at a frame rate of 100 Hz, and were recorded while gazing at specific dot patterns displayed on a blank screen with different target movements. Each recording, lasting approximately 5 min, consisted of a set of patterns: (a) Ring-shaped patterns at ±20 degrees eliciting smooth pursuit movements, at different depths; (b) Random point changes to induce saccades, where the targets were moved in a ±20 degree cone from 50 cm in depth to 600 cm. The background illumination was constant throughout each recording. The dataset was anonymized to remove any personally identifiable information on the subjects for further processing. Original recordings were later divided into one recording per subject and pattern used.

### 3.2. Gaze Prediction

**Figure 2:**
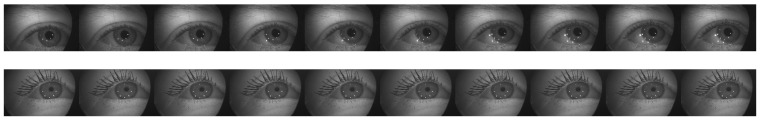
Example of saccadic (**top row**) and smooth pursuit (**bottom row**) eye movements during 100 ms.

This subset of data are aimed at fostering research on spatio-temporal methods for gaze estimation and prediction for tasks involving different eye movements, such as saccades, fixations, and smooth pursuit. Examples of different eye movements included in the dataset are illustrated in Figure 2. Two important variables to take into account in the design of a prediction-oriented dataset are: (1) the observable time window (i.e., the number of frames used to initialize a gaze prediction model); and (2) the prediction window (i.e., the number of frames to predict). Based on the average fixation time between 150 and 300 ms [37] and saccades generally being between 20 and 200 ms [38], we provide 50 frames (500 ms) as observable window and hypothesize that it is a reasonable number to set as the maximum number of frames that can be used to initialize a gaze prediction model. Furthermore, based on the eye movement characteristics [39] and the frame rate of our dataset, we propose to predict 1 to 5 frames (10 to 50 ms) into the future, which is a useful range in AR/VR applications [40].

#### 3.2.1. Dataset Curation

Two types of patterns were selected for this data subset, due to the differences in eye movement dynamics associated with them: (a) Ring-shaped smooth pursuit-elicited pattern, intertwining 1s fixations at a fixed depth with 1s-long smooth pursuit movements as smooth transitions between fixation points, with a total of 17 fixations and 16 transitions per recording; (b) Saccade-elicited pattern, with 1s-long randomly located target fixations at different depths and instantaneous (0.1 s) target transitions, with up to 20 fixations per recording. All subjects were recorded following both pattern types. The former pattern was the same for all subjects, while the latter was always different due to its random nature. However, note that although the ring-shaped pattern was the same, the elicited gaze trajectories were different across subjects due the subject-dependent nature of eye movement dynamics [41].

Frames with invalid ground-truth gaze vector, due to either subject distractions, blinks or incorrect detections (see Section 3.2.2), were manually discarded, maintaining 80 out of the initial 90 subjects. The remaining data were further processed by randomly selecting *s* non-overlapping sequences of *f* contiguous frames each per recording, with a maximum of 100 frames (1 s) per sequence. This way, each sequence can contain either fixations only, smooth pursuit movement only, a combination of fixation and smooth pursuit, or a combination of fixation and saccadic movement, depending on the pattern segment they fall within. Each eye was processed individually, and right eye (from the camera point of view) and respective ground-truth vectors were flipped horizontally to seamlessly augment the data. These processing steps also allowed to break the symmetry of the ring-shaped pattern, making it difficult to recover the original pattern shape. For the challenge, the type of pattern used for each sequence was not provided, to prevent challenge participants from using that prior information for their proposed gaze prediction methodologies.

We devised three different subject-independent partitions of our dataset, with 32 subjects for training, 8 for validation and 40 for test. To do so, we performed stratified sampling in terms of gender, ethnicity, age, and glasses, to ensure a representative sample for testing. The training subset consists of 10 100-frame sequences per eye, type of pattern, and subject, with a total of 4000 images per subject and 128,000 images in total. Assuming that we can use up to 50 frames to initialize a gaze prediction model to predict up to 5 frames into the future, using a sliding window of stride 1 allows us to obtain up to 46 subsequences in a 100-frame sequence, which sums up to 58,880 final sequences in the training set (i.e., 1280 100-frame sequences in which a sliding window of stride 1 and size 55 is used to create subsequences). For validation and test subsets, however, we chose to provide 55-frame sequences to have one set of initialization frames and predictions per sequence, which facilitates evaluation and analysis. To compensate for the difference in number of effective sequences with respect to the training set, we selected approximately 80 55-frame sequences per type of pattern and subject, with 70,400 images for validation and 352,000 for test. For subjects for which there was not enough valid data to obtain such 80 sequences per pattern, the maximum number of valid sequences was selected, and the remaining sequences were obtained from other subjects until we obtained the desired number of sequences, 1280 and 6400, respectively. Please note that the goal of training and validation subsets is both gaze estimation and prediction, while the goal of the test subset is gaze prediction. Therefore, even though the number of images is substantially bigger for the test set, the number of sequences is what we focus on.

**Figure 3:**
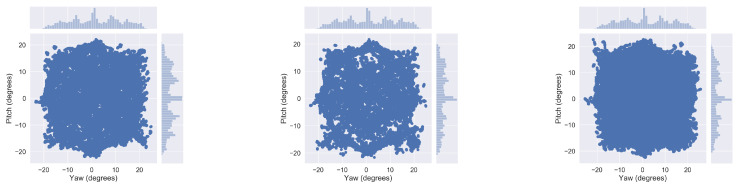
2D gaze angle distributions for train (**left**), validation (**center**) and test (**right**) splits of the Gaze Prediction data subset.

The distribution of ground-truth gaze angles (horizontal and vertical components of gaze) for each data split is depicted in Figure 3, and their general statistics in terms of subject variability and number of images and sequences per split shown in Table 2.

#### 3.2.2. Annotations

Each eye image was provided along with its respective ground-truth 3D gaze vector, corresponding to the visual axis, in the headset coordinate system (origin of coordinates located at the midpoint between the eye boxes of left and right eye, and oriented such that X-axis points left, Y-axis points up, and Z-axis points forward, from the person’s perspective). Gaze vectors and cornea centers of each eye were obtained using a hybrid model, which combines a deep eye-segmentation model (see Section 4.2) with a classical user-calibrated glint-based model [16], similarly to commercial eye trackers. Since these models are frame-based and thus may produce fluctuating estimates, a median filter of window size 5 was used to temporally smooth resulting gaze vectors. An offset correction was further applied to them per subject and pattern, by subtracting the average difference between cornea-to-target vectors and gaze vectors.

Besides the eye-image sequences and associated gaze vectors provided for the challenge, we are including additional metadata, namely: type of pattern used (saccade-based or smooth pursuit-based), 3D target coordinates, target status (i.e., if the target is static, moving, or just finished moving to allow for eye catch-up/readjustment), 3D point of gaze, 3D cornea center coordinates, internal (anonymized) subject ID, and sequence ID.

### 3.3. Semantic Eye Segmentation

This subset of data is aimed at exploiting the temporal information available in the form of short temporal sequences of eye images to improve the semantic segmentation accuracy achieved by treating each frame separately. Although there could be multiple approaches to exploit temporal information for improving semantic segmentation, we resort to a simple and practically useful problem of accurate label propagation from a few labeled images to all the frames in a sequence. On the one hand, this problem can serve as a testbed for future improvements in the latest deep-learning-based spatio-temporal models for real-time inference in videos, few-shot learning, geometry-constrained semantic segmentation, and/or co-segmentation. On the other hand, this problem also helps in generating high-quality pseudo-ground-truth data generation for training semantic segmentation networks.

#### 3.3.1. Dataset Curation

**Figure 4:**
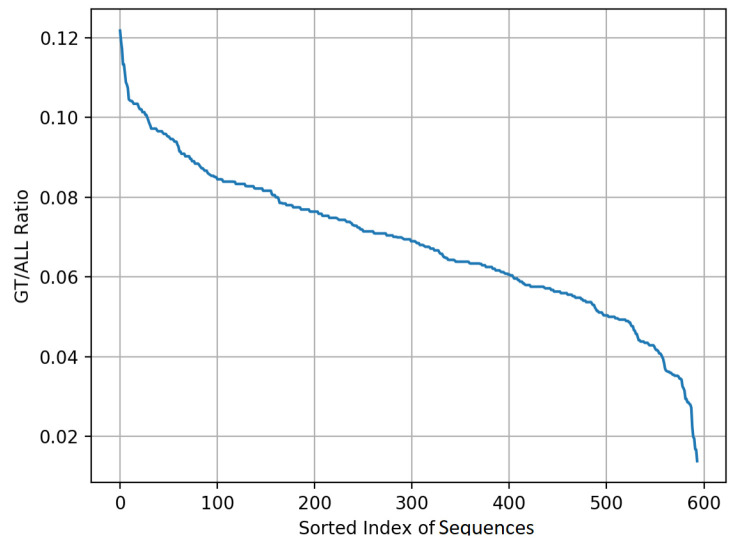
The ratio of labels vs. total number of samples for all the 594 sequences shown in the decreasing order.

We start the curation process with the initial data, i.e., a total of ∼600,000 images in the form of 594 temporal sequences and 11,476 hand-annotated semantic segmentation masks chosen randomly for annotation. All sequences belong to the ring-shaped pattern category, with target depths ranging from 50 cm to 600 cm. Let us define the *label-ratio*, *R*, as:

(3) $$R^{i}=\frac{N_{label}^{i}}{N_{total}^{i}}\times 100\%,$$

where $N_{label}^{i}$ and $N_{total}^{i}$ are the number of labeled samples and the total number of samples in the $i^{th}$ sequence $S_{i}$, respectively. For this dataset, we decided to set R≈5%, or in other words, provide ∼5% labeled samples for each sequence. This choice is motivated from the practical considerations of the size of the available dataset and annotations. To maintain R≈5% for training, we subsampled the data at 5 Hz and chose the top 200 sequences sorted in decreasing order of *R*, which resulted in ∼150 frames (∼30 s of recording) for a total of 29,476 frames with 2605 semantic segmentation annotation masks. Figure 4 shows the ratio of labels vs. total number of samples for all the 594 sequences. Out of the total 2605 annotations, we randomly hide 5 samples per sequence as the test samples, which eventually leads to R≈5% for training and 3% for testing. The 200 sequences were obtained from 74 different subjects. Additional statistics for this dataset can be found in Table 3. Please note that the statistics are presented at a complete sequence level and not at frame level.

#### 3.3.2. Annotations

**Figure 5:**
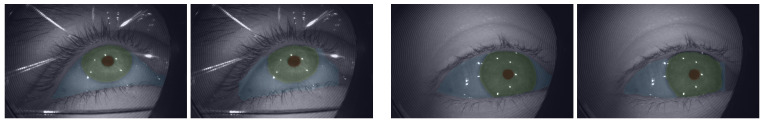
For each pair of images, examples of human annotations (**left**) and baseline model performance (**right**) for eye semantic segmentation (best viewed in color).

Human annotations are provided in the form of pixel-level segmentation masks with the following labels: (1) eye region, (2) iris, and (3) pupil. Annotation quality was evaluated by estimating the mIoU score of annotations, ensuring that no labels produced by at least two annotators have less than 80% mIoU. The right eye was flipped horizontally to align lacrimal caruncles across left and right eye annotations, so the annotators have consistency. The lacrimal caruncles were labeled as part of the eye region. In half occlusions, the annotations only include visible parts of the pupil and iris, and eyelashes are labeled as part of the underlying pupil or iris region. Finally, during blinks a thin sliver of the eye region is labeled. Examples of human annotations are provided in Figure 5.

Besides the eye images and associated segmentation masks provided for the challenge, we are also releasing the internal subject ID associated with each sequence, so that sequences corresponding to the same subject can be linked.

## 4. Baseline Methodologies

In this section, we describe and evaluate a set of baseline models for each data subset to demonstrate the usefulness and validity of the data for the suggested tasks.

### 4.1. Gaze Prediction

We propose a simple baseline model for the Gaze Prediction subset, in which spatio-temporal information is not jointly leveraged. Instead, we disentangle gaze estimation from prediction, estimating first the line of gaze from each eye image individually, and then performing gaze prediction based on the previously estimated gaze vectors.

First, we convert the ground-truth 3D gaze vectors to 2D spherical coordinates, representing yaw (horizontal component of gaze) and pitch (vertical component of gaze) angles. For gaze estimation, we train a deep-CNN based on a modified ResNet of 13 convolutional layers, as in [23]. The CNN backbone was coupled with a 32-hidden unit fully connected layer (FC) and a 2-hidden unit FC linear regression layer, to estimate 2D gaze angles. To compensate for the gaussian-like distribution of the data, we fit a multivariate Gaussian to the training set and weight the samples with their inverse probability. The network was trained end-to-end on 75% of the training data following such weighting scheme for 50 epochs with ADAM optimizer, initial learning rate of 0.001 and batch size of 32. For training, data were randomly augmented in terms of brightness, horizontal and vertical shifts, and rotation. Model training and inference was performed on downsampled images of 160 × 100 pixels. The total number of parameters of the model was 206K and the resulting model size is 828 KB. The trained gaze estimation network is tested on the validation set, obtaining an average angular error of 4.58 degrees, which is in line with state-of-the-art subject-independent approaches [24].

Our gaze prediction approach relies on linear regression. In particular, we use a window of 50 estimated gaze angles to compute the regression parameters using 2 independent regression models, i.e., one per gaze axis in 2D. The estimated parameters are used to predict the next 5 frames into the future. We apply our trained gaze estimation network on the test subset to estimate gaze for the first 50 frames of each sequence, compute linear regression parameters on them and predict the next 5 frames. Table 4 summarizes the obtained results. We can see that the error increases with time, which is expected. Furthermore, we can also observe that such a simple approach works fairly well for fixation and smooth pursuit sequences, which account for most of the dataset samples. However, the obtained error for saccades is large, as observed from p95 values. This is also expected mainly for two reasons. First, saccades usually follow a ballistic trajectory, thus not properly modeled with a linear model. Second, saccades have a duration of about 20 to 200 ms; therefore, linear regression cannot predict extremely short saccades that happen after the initialization window. We decided to include saccades in the challenge despite saccadic suppression to keep it generally applicable for uses cases where the suppression is not sufficient.

### 4.2. Semantic Eye Segmentation

To set a baseline for spatio-temporal eye-segmentation algorithms, we chose to train a deep-CNN on 1605 images whose corresponding hand-annotated semantic segmentation masks are provided. The network follows an encoder-decoder architecture loosely based on SegNet [42]. Modifications include a power-efficient version containing 7 downscaler blocks for the encoder and 7 upscaler blocks for the decoder. Each block contains separable convolution that factorizes depth-wise convolution and a 1 × 1 convolution to optimize for computation cost. We use LeakyReLU activation and multiplicative skip connections with guide layers that reduce the layers to a single channel to pass to the decoder, which again reduces computational cost.

We trained the network for 150 epochs with ADAM optimizer, with initial learning rate of 0.004 and batch size of 128. We used random rotation and intensity scaling for augmentations. Model training and inference was performed on downsampled images of size 128 × 128 pixels. The total number of parameters of the model was 40K and the resulting model size was only 300 KB.

The trained network is tested on the hidden set of test samples without exploiting any temporal information, achieving a mIoU score of 0.841 (see Table 5 for complete results).

## 5. Challenge Participation and Winning Solutions

The Gaze Prediction challenge received a total of 13 valid entries, while the Sparse Semantic Segmentation challenge received 22 valid entries. From them, the code implementations of the top-3 winning teams of each competition track were evaluated and the final winners announced at the workshop (See https://research.fb.com/programs/openeds-2020-challenge/, last accessed on 25 June 2021, for more details). What follows below is a brief description of the methods explored by the winning teams and the performance numbers produced.

### 5.1. Gaze Prediction Challenge Winners

In Table 6, we show the average gaze prediction error achieved by the top 3 winners, in comparison to the baseline model as described in Section 4.1.

All three top winning teams used a two-stage strategy to produce their winning solution. The first stage comprised of building a regression model to estimate the gaze vector per image for the sequence of training images. The output of the first stage was fed into a prediction network (or algorithm) to predict the future frames. Both team *random_b* and team *EyMazing* found the Resnet18 module [43] to work best to estimate per-image gaze vectors. Team *random_b* was able to optimize the estimation network using a hard data augmentation strategy, whereby for every image augmentation that involved rotation of the input image, the rotation matrix was also applied to the target gaze vector. Team *Eymazing*, on the other hand, relied on using standard data augmentation methods. However, they used a cosine loss function [44] applied to the normalized gaze vector coordinates, as opposed to the traditional mean-squared-error loss function used by team *random_b*. In contrast, team *Dmitrykonolov* used an EfficientNet-B0 backbone [45] to train their gaze vector estimation model coupled with a custom loss function derived from their prior work [46].

To train the prediction network, team *random_b* chose a rather simple strategy: check if there is a fast change in gradient of the gaze vectors in the last 7 frames of the sequence and if so, add the computed gradient linearly to the predicted frames, otherwise use moving average gaze vector derived from the last 3 frames. The proposed algorithm was applied independently to each axis of the gaze vector. Team *Eymazing* and team *Dmitrykonolov*, on the other hand, chose to train a standard Long-Short-Term Memory (LSTM) recurrent neural network [47] to predict the future gaze vector frames. Team *Eymazing*, again, used the cosine loss function for their LSTM network, computed only on the predicted frames, while team *Dmitrykonolov* relied on their pre-designed custom loss function.

The following general observations can be drawn from the experience of these three winning teams. First, training estimation and prediction networks separately. Second, overfitting was a significant challenge, especially when using complex backbone neural architectures for training the estimation network. As such, Resnet18 with suitable data augmentation methods or the choice of a suitable loss function worked best. Finally, a rather simple prediction algorithm, as proposed by team *random_b*, seemed to work best, producing their winning model. This team, however, noted after the competition was over that they were able to produce even better performance numbers using a LSTM recurrent network for training. This conforms with the findings by team *Eymazing* as well as team *Dmitrykonolov*.

### 5.2. Sparse Semantic Segmentation Challenge Winners

In Table 7, we show the mIoU scores produced by the top-3 winning teams for the Sparse Semantic Segmentation Challenge in relation to the baseline score.

The Sparse Semantic Segmentation challenge called for propagation of semantic labels in a video sequence of eye images. However, none of the winning teams produced a solution to leverage the temporal information in the video sequence of images. Instead, a common theme running across the solution developed by all 3 winning teams was the use of a rather simple encoder-decoder neural network architecture, extensive data augmentations, pseudo-labeling on unlabeled images and model ensemble. Specifically, the first-place winner, team *BTS Digital*, used a UNet segmentation network [48] to train a semantic segmentation model on a weighted combination of binary cross-entropy loss, focal loss [49] and dice loss [50]. Two rounds of pseudo-labeling [51] with 90 % of unlabeled data coupled with an ensemble of models trained on different cross-validation splits produced their winning entry. The second-place winner, team *tetelias*, on the other hand used a DABNet backbone [52] pre-trained on the Cityscapes dataset [53] for training the eye semantic segmentation model. Their winning solution heavily relied on advanced data augmentation methods such as GridMask [54] and FMix [55] followed by ensemble averaging on multiple trained network models. The third-place winner, team *JHU*, trained an ensemble of UNet models followed by heavy dose of pseudo-labeling on all the available unlabeled images.

In summary, contrary to our original motivation for seeking novel ideas to propagate knowledge from sequences of eye images to augment semantic segmentation for video frames with missing labels, pseudo-labeling with ensemble averaging proved to be the winning recipe to produce winning solution to the proposed problem. Although the winning numbers are impressive, we still believe that there is room for improvement on this task, should novel methods to leverage sequence information be devised.

## 6. Conclusions

In this paper, we have reviewed the OpenEDS 2020 Challenge motivation, baselines proposed, winning solutions and findings. Moreover, we have described the OpenEDS 2020 Dataset, a novel dataset of eye-image sequences consisting of up to 87 subjects of varied appearance performing different gaze-elicited tasks. The dataset consists of two subsets of data, one for each competition track of the challenge, i.e., one devoted to gaze prediction and another devoted to eye semantic segmentation, with the goal of fostering the exploitation of spatio-temporal information to improve the current state of the art on both tasks. Obtained results with proposed baseline methods and winning solutions, all of them based on deep learning techniques, demonstrate the usefulness of the data, and serve as a benchmark for future approaches coming from computer vision, machine learning and eye tracking fields.

In particular, the learnings from the outcome of the Gaze Prediction challenge helped us refine the formulation for the OpenEDS 2021 Gaze Prediction challenge (OpenEDS 2021 Eye Tracking Challenge https://research.fb.com/programs/facebook-openeds-2021-challenge/, accessed on 25 June 2021). Based on the observation that all winning teams for the OpenEDS 2020 Gaze Prediction challenge trained the estimation network first and then followed up with the training of the prediction network, the 2021 Gaze Prediction challenge is solely focused on the framing of the prediction problem. The time series of gaze is derived from individuals freely exploring two virtual environments participating in varying activities such as reading, drawing, shooting and object manipulation [56]. The challenge calls for predicting the gaze using the trajectory of available gaze data. We equally anticipate that the findings from the 2020 Challenge will spur innovative solutions for the OpenEDS 2021 Gaze Prediction challenge.

## Figures and Tables

**Table 1:** Summary of existing, publicly available eye tracking datasets containing sequences of real eye images obtained using IR cameras. “Illum.”, illumination; “Res. WxH”, image resolution width × height; “Freq.”, sampling rate; “# Subj.”, number of subjects; “Head mov.”, head movement allowed during recordings; “#. Seqs.”, number of sequences.

Name	Camera Viewpoint	Illum. Type	Freq. (Hz)	Res. W × H (pixels)	# Subj.	Head mov.	# Seqs.	Annotations	Tasks
PoG [29] (2012)	On-axis (1 eye)	1 (indoors)	29.97	720 × 480	20	Y	6 per subj.	Target pixel, 3D monitor and headset locations	Elicited (fixations and smooth pursuit)
LPW [30] (2016)	On-/off-axis (1 eye)	Continuous (indoor/ outdoor)	95	720 × 480	22	?	3 × 20 s per subj.	2D pupil position, segment. masks (provided by [31])	Elicited
NVGaze [32] (2019)	On-/off-axis (2 eyes)	Constant/ varying (indoors)	120	640 × 480	30	N	56	2D gaze direction, pupil position, blink labels	Elicited (fixations)
GW [33] (2020)	Off-axis (2 eyes)	Continuous (indoor/ outdoor)	120	640 × 480	19	Y	9 min. per subj.	Eye mov. types, 3D gaze vector, head pose	4 real-world tasks
Multimodal Eye Movement [34] (2021)	Off-axis (1 eye)	Continuous (indoor/ outdoor)	25	192 × 144	19	Y	30 min. per subj.	Eye mov. types, eye params., segment. masks ([35]), optical vectors ([36])	Car ride (real and simulated)
Ours (OpenEDS 2020)	On-axis (1 eye)	1 (VR)	100	640 × 400	87	N	∼9160	3D gaze vector, target, point of gaze, cornea center, segment. masks	Elicited (fixations, saccades, smooth pursuit)

**Table 2:** Statistics of the gaze prediction data subset.

	Gender		Ethnicity			Age				Accessories		Number of	
	Female	Male	Asian	Caucasian	Other	21–25	26–30	31–40	41+	Glasses	Makeup	Images	Seqs.
**Train**	9	23	10	15	7	6	7	13	6	8	5	128K	1280 (×46)
**Val.**	3	5	1	4	3	2	1	3	2	2	0	70.4K	1280
**Test**	12	28	16	17	7	10	10	14	6	11	5	352K	6400
**Total**	24	56	27	36	17	18	18	30	14	21	10	550.4K	8960 (66,560)

**Table 3:** Statistics of the subjects featured in the 200 selected sequences for the eye-segmentation subset.

Gender		Ethnicity			Eye Color				Age				Accessories	
Female	Male	Asian	Caucasian	Other	Brown	Blue	Hazel	Green	21–25	26–30	31–40	41+	Glasses	Makeup
27	47	31	30	13	50	14	4	6	17	15	25	16	21	14

**Table 4:** Baseline performance for gaze prediction, reported in terms of Prediction Error (PE) between predicted and ground-truth 3D gaze vectors, in degrees. p50, p75, and p95 scores denote the 50th, 75th, and 95th percentile scores of the PE${}_{t}$ distribution, respectively.

Time Step	PE${}_{t}$	p50	p75	p95
1 (10 ms)	5.28	4.56	6.73	11.89
2 (20 ms)	5.32	4.57	6.79	11.99
3 (30 ms)	5.37	4.61	6.83	12.13
4 (40 ms)	5.41	4.63	6.87	12.30
5 (50 ms)	5.46	4.65	6.92	12.48
Average	5.37	4.60	6.83	12.16

**Table 5:** Baseline performance (mIoU) for sparse semantic segmentation without the use of temporal information.

Background	Sclera	Iris	Pupil	Average
0.971	0.674	0.882	0.835	0.841

**Table 6:** Performance numbers from the top-3 winning entries to the OpenEDS 2020 Gaze Prediction challenge, reported in terms of Prediction Error, in degrees.

Model	PE
1st place winner (team *random_b*)	3.078
2nd place winner (team *EyMazing*)	3.313
3rd place winner (team *DmitryKonolov)*	3.347
Baseline	5.368

**Table 7:** Performance (mIOU) from the top-3 winning entries to the OpenEDS 2020 Sparse Semantic Segmentation Challenge.

Model	mIOU Score
1st place winner (team *BTS Digital*)	0.9517
2nd place winner (team *tetelias*)	0.9512
3rd place winner (team *JHU*)	0.9502
Baseline	0.841

## Data Availability

The OpenEDS2020 Dataset, described in this paper, can be accessed upon request by sending an e-mail to openeds2020@fb.com.
